# Integrating quarry dust and industrial waste in producing eco-friendly hybrid geopolymer concrete

**DOI:** 10.1038/s41598-025-28913-7

**Published:** 2025-12-31

**Authors:** Ahmed AL-Mowafy, Mohamed E. El-Zoughiby, Osama Youssf

**Affiliations:** 1 Structural Engineering Department, Mansoura University, Mansoura, Egypt; 2 Civil Engineering Department, Horus University-Egypt, New-Damietta, Egypt

**Keywords:** Hybrid geopolymer concrete, Curing methods, Fire resistance, Mechanical properties, Microstructure, Engineering, Environmental sciences, Materials science

## Abstract

This paper proposes the production and testing of hybrid geopolymer concrete (HGC) as a sustainable substitute for the well-known traditional slag- and fly ash-based geopolymer concrete. A total of fourteen sustainable HGC were proposed with a variety of quarry dust materials such as granite powder (GP), basalt powder (BP), and dolomite powder (DP) as partial substitutes of fly ash (FA). Additionally, industry waste materials namely, plastic shales (PS), plastic pellets (PP), and crumb rubber (CR) were used as partial substitutes of sand in the proposed HGC. Several variables were investigated including geopolymer binder type, concrete curing methods, and mixing procedures. Heat followed by water (HW) and heat followed by air (HA) curing methods were applied on the proposed HGC. Workability, compressive strength (under ambient and high elevated temperature), splitting tensile strength, and flexural strength were the physical, and mechanical properties measured. The measured properties of HGC were also compared with those of equivalent cement-based concrete mix. Selected HGC mixes were further analyzed using scanning electron microscopy (SEM) and energy dispersive X-ray (EDX) spectroscopy. The results indicated that the proposed HGC is a practical and eco-friendly substitute of both traditional geopolymer and cement-based concrete, as it showed similar or better performance. The compressive strength increased by about 10% when 15% of the FA was replaced by BP or DP. Exposing the proposed concrete to high elevated temperature of 300 °C for 2 h increased its compressive strength by 20.5% for the control mix, 29.9% for mix contained 15% GP, 2.6% for mix contained 15% BP, 3.4% for mix contained 15% DP, and by 2.4% for mix contained 25% CR. However, all mixes lost strength when exposed to 600 °C for 2 h. Except for the Control mix, GP mixes, and PP mixes, the HW curing method showed lower strength in all mixtures. All HGC mixes showed better performance than the cement-based concrete. Microstructural analyses showed a thick and even structure for the HGC mixes, supporting their relatively high strength. This study demonstrates the substantial potential of HGC as a revolutionary concrete type for construction that coincides with global sustainability goals and meets contemporary building demands.

## Introduction

 Due to the current rapid global population increase and the construction of high-rise structures with the required infrastructure, Portland cement (PC) concrete is the most widely used building material on earth^1,2^. The present output of PC accounts for 5–7% of world carbon dioxide emissions (CO₂)^3–7^. Regular PC manufacture raises various environmental issues, including high energy consumption, the use of non-renewable raw resources, and significant CO₂ emissions^8–11^. Geopolymer concrete (GC) is a cement-free alternative to traditional cement-based concrete that utilizes industrial by-products abundant in silica and alumina, including slag and fly ash (FA). GC utilizes six times less energy and generates nine times less CO₂ in comparison to cement-based concrete^12^. GC also exhibits outstanding mechanical characteristics, minimal shrinkage, good durability, elevated fire and heat resistance, and improved resistance to sulfate and chloride attack.

GC made from FA is the most often used GC type. Though it requires high elevated temperature to cure well (60–120 °C for 24 h), it has been thoroughly investigated and shows numerous advantages like excellent workability, great strength, and less shrinkage (compared to slag-based GC)^13–17^. FA lacks calcium: hence, it has low reactivity and increases the concrete setting time while, if cured at ambient temperature, diminishing the early age strength^18^. Therefore, precast and heat-cured concrete members primarily use FA-based GC. To overcome the disadvantages of FA-based GC, calcium-rich materials are used, such as slag (a byproduct of producing iron and steel), which has been demonstrated to speed up the polymerization process^19,20^. Slag has been used as 50% of the binder material in air curing circumstances to produce geopolymer mortar with high strength^21^. A high level of hydration leads to self-desiccation, which creates high capillary pressure. This is why slag-based GC shrinks a lot more than FA-based GC^22^. Similar to that of FA, heat curing can increase slag GC’s early strength, but has a negative impact on its later age strength when compared with slag-based GC cured at ambient temperature^23^. Despite extensive investigations on traditional GC utilizing various binders, including industrial byproducts, GC has not yet gained widespread acceptance in building as a replacement for PC.

Granite powder (GP) trash, which is a byproduct of making granite, has gotten a lot of attention because of the environmental problems that come with throwing it away. Generally, GP waste is managed through a landfill or open dumps, both of which pose significant environmental risks. Fine particulates can permeate water and soil, resulting in the degradation of soil. and pollution^24,25^. Furthermore, GP waste can make the air more polluted and put nearby households at risk of breathing problems. Researchers found that utilizing GP waste in concrete as a substitute for cement and sand solves problems with disposal while simultaneously decreasing the demand for these materials. Because of the unique physical and chemical properties of the powder, adding GP waste to concrete improves its mechanical properties^25–27^. Basalt powder (BP) waste, a large amount of which is produced by basalt quarries, could be used again as a mineral additive in concrete, which could help clean up the environment and lower the health risks that associated with it^28,29^. An experiment by Celik et al.^30^ showed that using 30% BP with concrete as replacement of PC, enhanced its workability and late-age compressive strength. Likewise, a research study conducted by Uncik and Kmecova^31^ it was reported that the workability and strength of concrete were enhanced using BP as a mineral additive. Basalt powder (BP) has been effectively employed in the production of self-compacting concrete and ultra-high-performance concrete^28,29^. When dolomite rock is extracted and produced, it is ground and processed to create dolomite powder (DP) waste, an industrial byproduct. The effective use of DP can lower building expenses while enhancing the strength and durability of structures^32^. Using DP in concrete instead of cement has been shown to be beneficial in several studies. This has led to less waste going to landfills, less energy being used to make PC, lower CO₂ emissions, and reduced costs^33^. Kumar et al.^32^ investigated the impact of finely powdered dolomite on the essential mechanical and physical characteristics of concrete. They found that the compressive strength and flexural strength were noted as maximum when the cement replacement existed at 15% by DP.

Scrap tires represent a major and challenging stream of waste in modern society, mostly due to their durability and the large quantities discarded annually^29,34,35^. The incorporation of recycled scrap tires in concrete was initiated approximately 30 years ago to mitigate its negative environmental^2,36,37^. For example, in Australia more than 50 million car tires were past their useful lives in 2014. Just 5% of that quantity was recycled domestically, 32% was exported abroad, and 16% was dumped in landfills. Users randomly disposed of the remaining end-of-life tires at unidentified or unconfirmed locations^38,39^. It results in a few environmental issues^40,41^. Dumping tires in saturated soils can result in the leaching of toxic substances from their composition into groundwater, posing a significant environmental hazard^42,43^. The use of tire rubber in concrete as a partial substitute for mineral aggregates has been the subject of increasing investigation recently. This has led to the establishment of crumb rubber (CR) concrete^36,44^. Concrete aggregates are one natural resource that can be preserved through recycling tire rubber waste^45,46^. , particularly, the overuse of natural sand in industry and building is causing its cost to rise.

The issue of plastic waste (PW) pollution has spread throughout the world. The Association of Plastic Manufacturers (Plastics Europe) estimates that 370 million metric tons of plastic are produced annually at the present time^47^. Around the world, only 9% of PW is recycled; the remaining 80% is either landfilled or scattered over terrestrial and aquatic environments^48,49^. In nature, PW breaks down into microplastics (1 μm − 5 mm) and nanoplastics (1 –1000 nm) due to physical abrasion, ultraviolet radiation, changes in temperature, and biological metabolism^50^. Furthermore, the atmosphere has the capacity to carry microplastics and nanoplastics particles up to 95 km^51^, accessing the most isolated areas can significantly impact the overall air quality. The bad effects of microplastics and nanoplastics in the air are made worse by the fact that they can pick up and carry other environmental pollutants, such as heavy metals and organic contaminants^52,53^. PW is a big problem for the environment because it pollutes the air and water with tiny particles and leaks dangerous chemicals used to make polymers, such as solvents, initiators, catalysts, plasticizers, surfactants, lubricants, antioxidants, colorants, and flame retardants^54–56^. All of these could have negative effects on human and animal health, including cytotoxicity, hypersensitivity, acute reactions, and unintended immunological responses^47^.

Currently, research on the combined application of GP waste, BP waste, DP waste, CR, and PW in the production of GC to produce hybrid geopolymer concrete (HGC) remains limited. The objective of this research is to address this research gab by investigating the mechanical properties of HGC containing GP, BP, and DP as partial replacements of GC binder (FA) at volumetric levels of 15% (low), 30% (medium), and 45% (high), as well as CR and PW as a 25% of partial substitution for GC sand. The aim is to study how variations at different levels affect mechanical properties, ensuring that the high level does not exceed 50% to prevent negative impacts on the concrete performance. Two different forms of PW were used: plastic shales (PS) and plastic pellets (PP). The performance of the introduced HGC was assessed through several measurements including compressive strength, splitting tensile strength, and flexural strength. The compressive strength was measured under various curing conditions such as heat followed by air (HA) and heat followed by water (HW), as well as under high elevated temperatures (room temperature, 300 ᵒC, and 600 ᵒC). The microstructure of selected HGC mixes was analyzed using scanning electron microscopy (SEM) and energy dispersive X-ray (EDX) spectroscopy. The results will support further efforts to reduce carbon footprints in the building industry while also encouraging urban planning innovation.

This study distinguishes itself from existing research on binary and ternary geopolymer blends by introducing a comprehensive hybrid geopolymer concrete (HGC) formulation that integrates a wider variety of industrial by-products simultaneously—specifically, quarry dusts (GP, BP, DP) as partial FA substitutes, combined with various plastic wastes (PS, PP) and CR as partial sand replacements. Unlike prior works that typically focus on one or two supplementary materials, our approach systematically evaluates the synergistic effects of multiple waste materials on mechanical performance, and microstructure under different curing conditions and elevated temperatures. The unique scientific contribution lies in demonstrating that this multi-component HGC not only matches or surpasses the strength of traditional geopolymer and Portland cement concretes but also offers enhanced fire resistance and sustainability benefits. Practically, this broad integration of diverse industrial wastes supports circular economy principles by addressing multiple environmental challenges simultaneously, providing a scalable pathway for eco-friendly construction materials aligned with global sustainability goals.

## Experimental program

### Materials

**Fig. 1:**
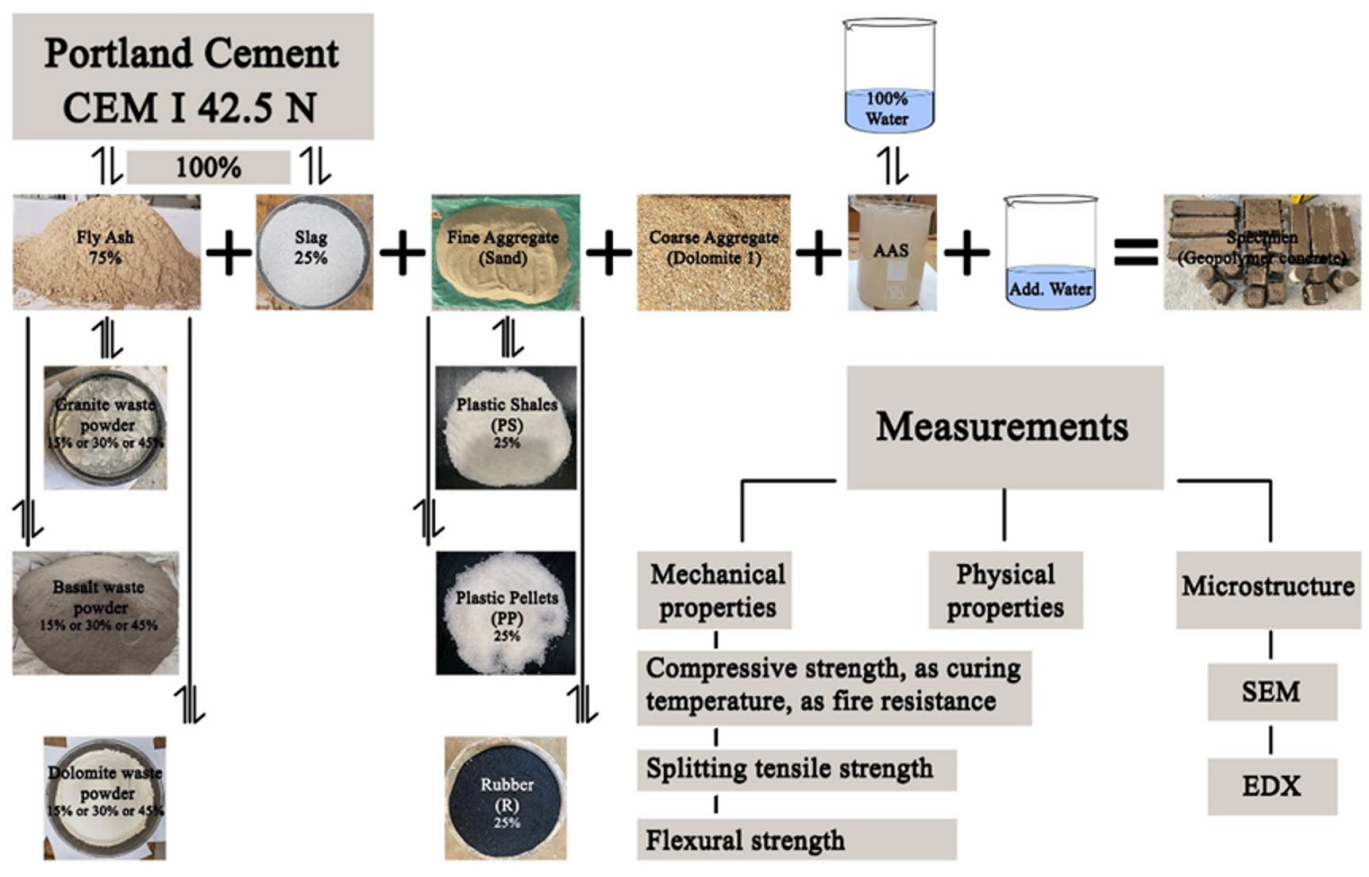
Experimental program flowchart for the current investigation.

**Fig. 2:**
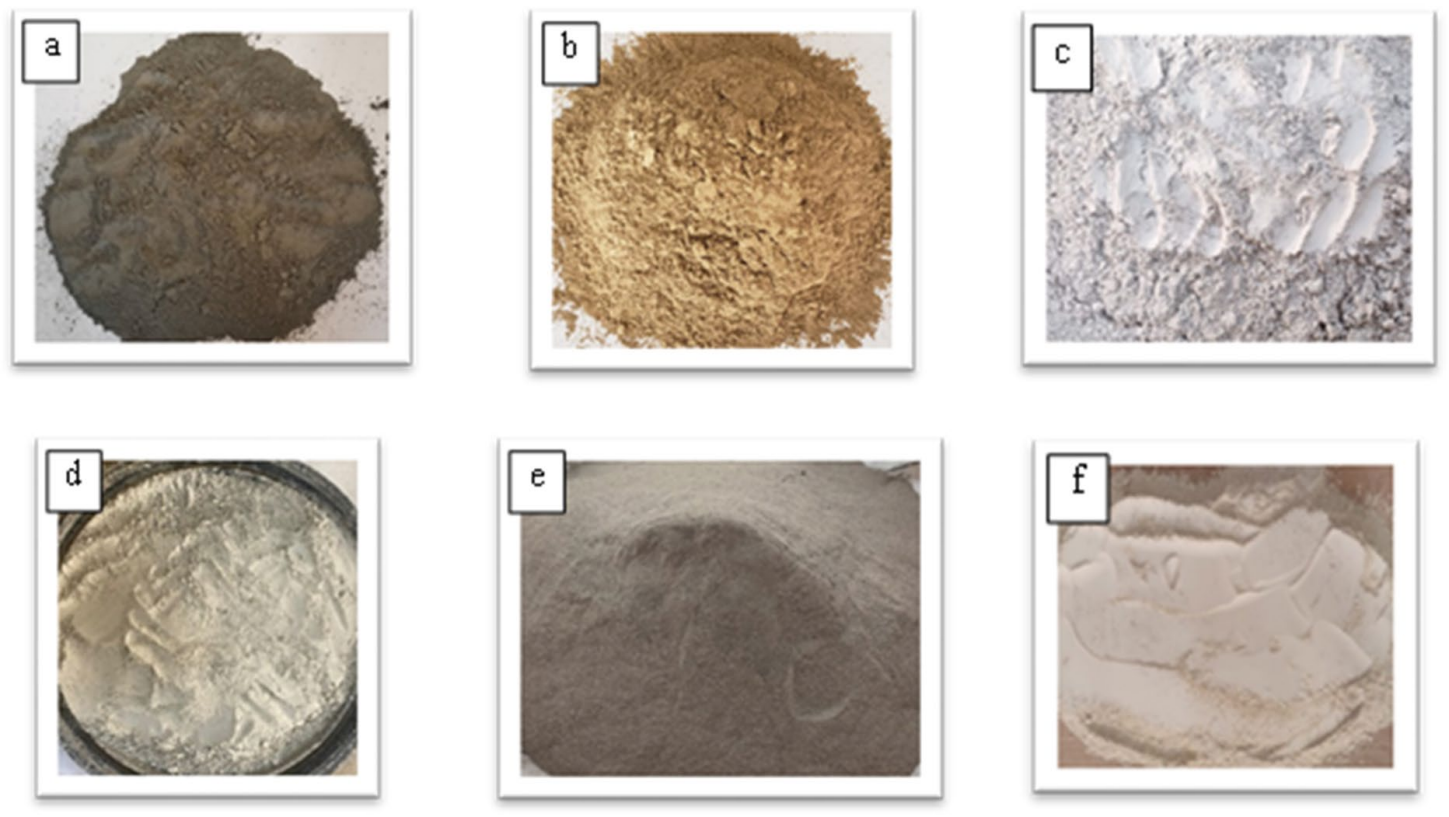
Geopolymer binders were used in this investigation: (**a**) CEM I 42.5 N, (**b**) FA, (**c**) Slag, (**d**) GP, (**e**) BP, and (**f**) DP.

**Fig. 3:**
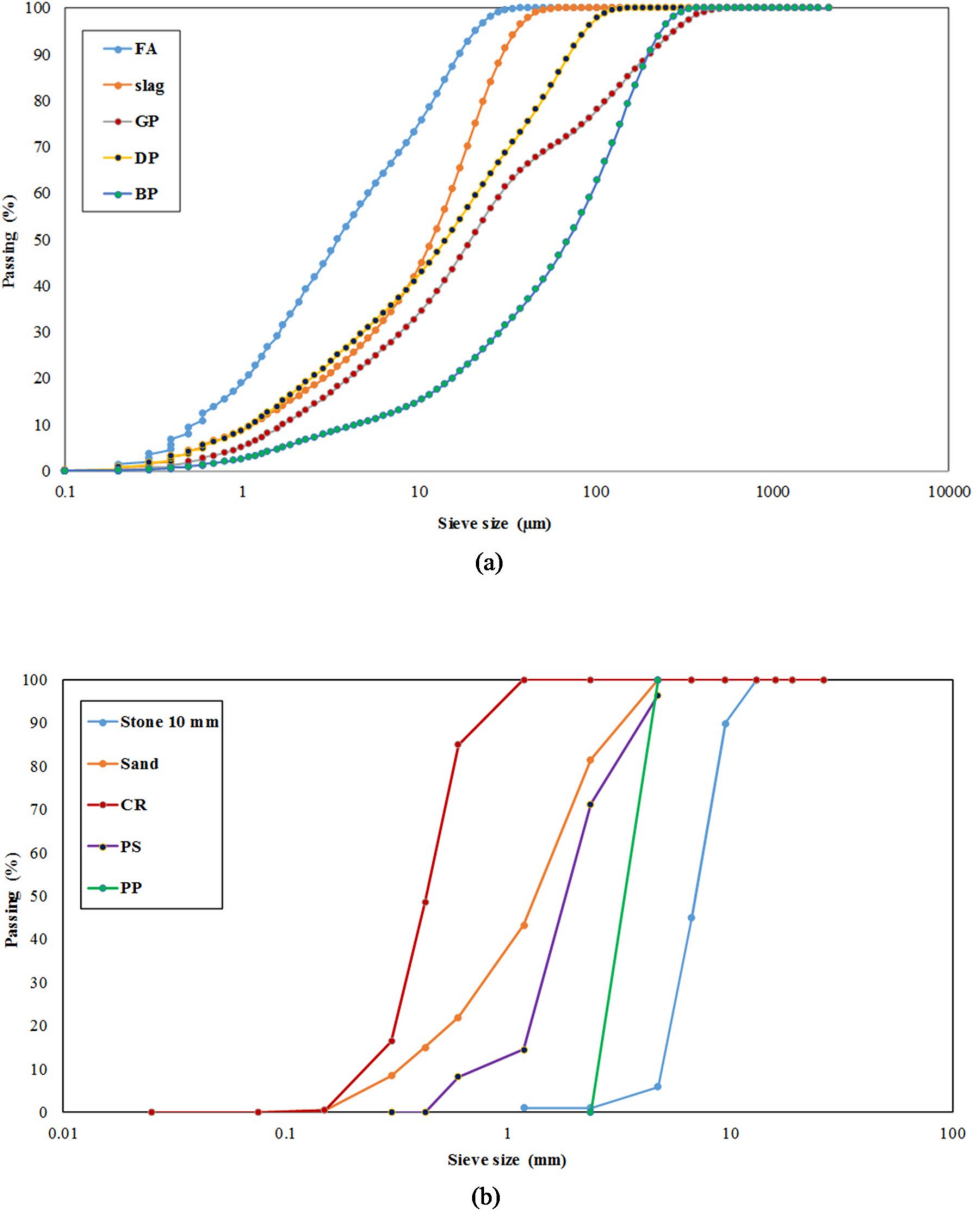
Distribution of particle size for the binders and aggregates utilized. (**a**) Binders, (**b**) Aggregates.

Figure 1 provides a summary of the materials utilized., measurement techniques, and testing processes followed in this study’s research methodology. This study investigated a total of fourteen mixtures. The control mix included FA and slag as binders at 75% and 25% contents, respectively. According to ASTM C618-15^55^, the FA is classified as Class-F when the total SiO₂, Al₂O₃, and Fe₂O₃ surpasses 70%. The content passing through 45 μm and the specific gravity were 87% and 2.57, respectively for FA, and 99% and 2.90, respectively for slag. GP, BP, and DP, which had specific gravities of 2.65, 2.95, and 2.84, respectively, were used to partially replace FA by volume at 15%, 30%, and 45% levels. Cement with a specific gravity of 3.15 was also used as the binder of a cement-based concrete mix for comparisons. Cement properties were carried out as Egyptian Standard Specifications ESS 4756–1/2013^61^. Figure 2 displays the binder materials utilized. Tables 1 and 2 show the chemical and physical compositions of the geopolymer binders used in this study. Figure 3(a) shows the distribution of particle sizes for all binders used.

10 mm dolomite stones with a specific gravity of 2.71 and unit weight of 1590 kg/m^3^, fineness modulus of 2.2 were used as the coarse aggregate. The fine aggregate was made out of 5 mm sand from a local quarry that had a specific gravity of 2.61 and unit weight of 1420 kg/m^3^, fineness modulus of 2.2. Fine CR with particle sizes ranging from 2.36 to 4.75 mm and a fineness modulus of 4.85, and unit weight of 530 kg/m^3^, PS with particle sizes ranging from 0.30 to 4.75 mm, and PP with particle sizes ranging from 2.26 to 4.75 mm to partially substitute the volume of concrete sand, which had specific gravities of 0.97, 0.90, and 0.90, respectively. Figure 3(b) illustrates the distribution of particle size for the aggregates employed in this study.

**Table 1:** Chemical compositions of the utilized geopolymer binders (%).

Geopolymer binder (%)	CaO	SiO_2_	Al_2_O_3_	Fe_2_O_3_	SO_3_	Na_2_O	K_2_O	MgO	*P*_2_O_5_	MnO	TiO_2_
PC	62.70	20.20	6.00	3.30	2.20	-	-	2.00	-	-	-
FA	5.80	51.10	18.10	9.70	1.00	3.94	1.84	7.30	0.20	-	-
Slag	29.20	39.98	16.22	1.11	2.25	1.04	0.65	7.74	0.01	0.491	0.61
GP	3.00	69.50	14.50	3.01	0.19	3.46	4.29	0.64	0.08	-	0.37
BP	11.36	47.52	15.49	14.03	0.09	2.11	0.87	3.97	0.51	0.26	2.11
DP	92.04	1.52	0.811	2.69	0.002	-	0.129	2.495	0.284	-	-

**Table 2:** Physical characteristics of all utilized geopolymer binders.

Characteristics	CEM I 42.5 *N*	FA	Slag	GP	BP	DP
Specific gravity (g/cm^3^)	3.15	2.57	2.90	2.65	2.95	2.84
Specific surface area (cm^2^ /g)	-	26,700	6000	8000	5300	1700
Chloride (Cl)	-	-	0.30	0.11	-	1.01
Loss of ignition (L.O.I)	1.70	0.20	-	0.65	1.34	44.07

In HGC mixtures, an alkaline solution is used as the activator and possessed a specific gravity of 1.28. Sodium hydroxide (SH) and sodium silicate (SS) solutions were combined for making the alkaline solution. Hassan et al.^13^ demonstrated in their review study that the optimal weight mixing ratio of those solutions ranged from 1.5:1 to 2.5:1 (SS: SH). A weight mixing ratio of 1.7:1 (SS: SH) was chosen and utilized in this study to facilitate the preparation of the necessary specimens. An alkaline solution was prepared 24 h before using it in concrete mixing. A 12 M concentration of SH was created by dissolving 480 g of SH granules in 1 L of water. Alzeebaree et al.^62^ determined that a 12 M SH concentration yields optimal mechanical and durability performance for geopolymers in comparison to 8, 10, 14, and 16 M concentrations. The SH solution was permitted to cool down for 2 h to avert clumping upon incorporation into the mixture. The SS solution was subsequently mixed with the SH solution, and the amalgamation was agitated for 20 min to achieve uniformity. The SH solution increased setting time and diminished mechanical strength, necessitating the incorporation of the SS solution to enhance the concrete’s performance. However, the concrete became softer and took two days longer to set after pouring when the SS solution was used alone^63,64^. The SS solution contained 28.6% SiO₂ and 8.9% Na₂O, had a molecular weight of 122.1 g/mol, and had a total solids content of 37.5%.

### Mix designs and mixing procedures

Mixtures of concrete were formulated with constant ratios of alkaline activator while FA was partially replaced by different binder materials at different replacement levels, as detailed in Table 3. The binders used in the control mix were slag and FA, whereas the nine mixtures having code started with G, B, or D incorporated GP, BP, and DP as partial replacements of FA at 15%, 30%, and 45% levels by volume. Mixtures PS, PP, and CR incorporated PS, PP, and CR as a 25% volumetric partial substitution of sand. All partial replacements were carried out by volume due to the differences between the specific gravities of the original materials and the substitutions, see section “Materials”. This was done to ensure a constant absolute volume of all comparable mixes of one cubic meter. Additionally, mixture PC was made with Portland cement with the same binder content of the control mix for comparisons. In both control and PC mixtures, the total amount of binder materials was set at 515 kg/m³, this mount varies with the replacement ratios of FA with other substituted materials, as detailed in Table 3, and the content of alkaline activators was set at 206 kg/m³. This kept the binder-to-alkaline activator ratio at 0.40 in all mixes. Extra water of 32 kg/m³ was utilized in all mixes.

All dry aggregates were mixed for one minute, followed by the addition of ½ water and another minute of mixing, a two-minute pause, and then the remaining ingredients and another two minutes of mixing to create the PC mixture. The following mixing techniques for traditional Portland cement concrete were comparable to those used for HGC mixes: Mix all dry aggregates for one minute, then add all free water and mix for another minute. Stop mixing for two minutes, then add the geopolymer dry binders and mix for three minutes, then add the activator gradually for the last two minutes.

**Table 3:** Concrete mixture ingredients (kg/m^3^).

Mix code	FA	Slag	GP	BP	DP	Cement	Sand	PS	PP	CR	Dolomite	AA	Water
Control	386	129	-	-	-	-	814	-	-	-	814	206	32
GP15	328	129	60	-	-	-	814	-	-	-	814	206	32
GP30	270	129	120	-	-	-	814	-	-	-	814	206	32
GP45	212	129	179	-	-	-	814	-	-	-	814	206	32
BP15	328	129	-	67	-	-	814	-	-	-	814	206	32
BP30	270	129	-	133	-	-	814	-	-	-	814	206	32
BP45	212	129	-	200	-	-	814	-	-	-	814	206	32
DP15	328	129	-	-	64	-	814	-	-	-	814	206	32
DP30	270	129	-	-	128	-	814	-	-	-	814	206	32
DP45	212	129	-	-	192	-	814	-	-	-	814	206	32
PS	386	129	-	-	-	-	611	70	-	-	814	206	32
PP	386	129	-	-	-	-	611	-	70	-	814	206	32
CR	386	129	-	-	-	-	611	-	-	76	814	206	32
PC	-	-	-	-	-	515	814	-	-	-	814	-	238

FA: fly ash, GP: granite powder, BP: basalt powder, DP: dolomite powder, PS: plastic shales, PP: plastic pellets, CR: crumb rubber, AA: alkaline activator.

### Specimen Preparation

**Fig. 4:**
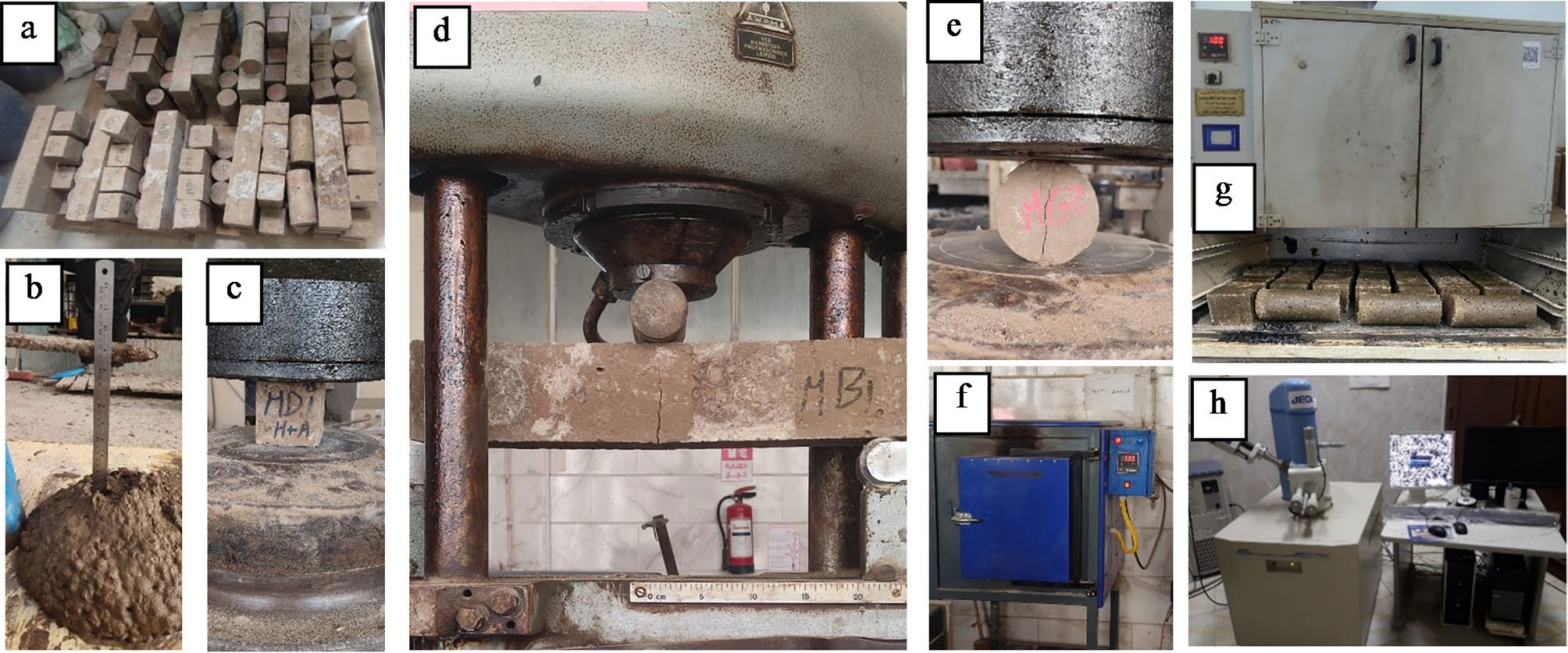
Test images: (**a**) specimens after casting, (**b**) slump test, (**c**) compressive strength test, (**d**) flexural strength test, (**e**) splitting tensile strength test, (**f**) elevated temperature oven, (**g**) curing oven, (**h**) and microstructure assessment.

The specimens that were prepared underwent testing to determine the effects of different variables on their physical, mechanical, and microstructural properties. Figure 4 displays the tests conducted for the proposed HGC mixes. The concrete workability was measured according to AS 1012.3.5^65^, as shown in Fig. 4(b). In accordance with BS 1881: Part 116–2004^66^, To evaluate compressive strength at 7, 28, and 56 days, three standard cube specimens (100 × 100 × 100 mm) were created for each measurement day.as shown in Fig. 4(c). At 28 days of concrete age, flexural strength was measured using prisms of 100 × 100 × 500 mm, in accordance with ASTM C78-16^67^, as shown in Fig. 4(d). Following the steps in ASTM C496/C496M-17^68^, the splitting tensile strength was measured after 28 days using the indirect splitting test, as shown in Fig. 4(e). The effect of high elevated temperature (300 °C and 600 °C) on concrete compressive strength at 28 days was measured using three standard cubic specimens (100 × 100 × 100 mm) per measure, as shown in Fig. 4(f).

The influence of curing conditions on the compressive strength of HGC was assessed using three cube specimens measuring 100 × 100 × 100 mm per mixture. The primary curing condition applied was the heat curing followed by air curing (HA), wherein molds containing fresh concrete cubes were maintained at a constant temperature of 23 °C for 2 h before being relocated to an oven set at 100 °C for a duration of 22 h. The hardened concrete cubes were thereafter placed in the ambient environment immediately after mold removal until the testing day. The other curing condition involved heat followed by water (HW), wherein molds containing fresh concrete cubes were maintained in an environment at a stable temperature of 23 °C for 2 h, thereafter, moved to an oven set at 100 °C for 22 h. The hardened concrete cubes were thereafter placed in a water bath immediately after mold removal until the testing day. Figure 4 (g) illustrates the specimens within the processing furnace.

EDX analysis and SEM imaging were utilized to assess the microstructure of selected HGC mixtures and to look at the elemental compositions of each proposed geopolymer concrete mix after 28 days. As shown in Fig. 4(h), the microstructure assessment was accomplished using a Jeol jsm 6510 LV device for SEM and Oxford X-max 20 device for EDX. The samples used for the microstructure analyses were taken from relevant specimens tested under compression.

## Results and discussions

The performance of the introduced HGC was assessed through several measurements including compressive strength, splitting tensile strength, and flexural strength. The compressive strength was measured under various curing conditions (HA and HW), as well as under high elevated temperatures (room temperature, 300 ᵒC, and 600 ᵒC). Table 4 shows the results of all measured properties, and Table 5 shows the standard deviation of these properties.

**Table 4:** Measured properties of HGC.

Mix Code	Slump (mm)	Unit Weight (kg/m^3^)	Compressive Strength (MPa)						Flexure Strength (MPa)	Splitting Tensile Strength (MPa)
			7 days	28 days		56 days	High elevated temperature 28 days (HA curing)		28 days	28 days
			HA	HA	HW	HA	300 °C	600 °C	HA	HA
Control	250	2500	60.0	60.2	63.2	65.8	72.5	30.3	8.5	4.4
GP15	250	2588	55.5	60.6	63.3	68.5	78.8	26.4	9.0	4.4
GP30	200	2532	49.0	50.8	53.8	57.1	48.0	25.1	7.6	4.1
GP45	180	2526	46.0	50.3	52.1	50.7	46.4	21.0	6.2	4.1
BP15	250	2560	67.4	66.3	60.0	76.3	68.0	50.7	10.0	4.9
BP30	180	2527	67.3	64.1	59.4	72.7	54.4	36.1	8.6	4.6
BP45	145	2513	57.0	53.5	49.3	57.2	48.9	32.7	6.6	4.14
DP15	210	2547	83.7	66.3	55.8	76.4	68.5	47.6	10.0	4.8
DP30	200	2530	75.0	61.5	54.7	71.4	54.3	45.2	7.7	4.6
DP45	190	2524	72.3	55.0	52.9	60.5	53.6	32.4	6.6	4.2
PS	215	2464	42.7	47.4	34.5	48.8	32.7	10.6	5.7	3.3
PP	40	2453	44.3	44.1	54.1	45.4	30.5	10.6	5.4	3.0
CR	170	2491	37.5	36.9	43.9	43.2	37.8	14.4	7.3	3.9
PC	150	2412	23.7	24.9	24.9	23.4	21.8	19.8	4.8	1.0

**HA**: Heat curing followed by air curing, **HW**: Heat curing followed by water curing.

**Table 5:** Standard deviation of measured HGC properties.

Mix Code	Unit Weight (kg/m^3^)	Compressive Strength (MPa)						Flexure Strength (MPa)	Splitting Tensile Strength (MPa)
		7 days	28 days		56 days	High elevated temperature 28 days (HA curing)		28 days	28 days
		HA	HA	HW	HA	300 °C	600 °C	HA	HA
Control	50.0	1.4	3.4	1.1	8.2	3.5	1.3	0.5	0.3
GP15	10.5	2.1	2.1	1.8	0.7	1.1	3.1	0.2	0.1
GP30	13.9	2.8	2.2	0.8	2.5	2.8	2.6	0.3	0.1
GP45	24.1	1.4	0.2	1.6	1.2	0.8	0.7	0.3	0.1
BP15	3.3	2.2	0.7	3.8	0.6	2.8	3.8	0.4	0.2
BP30	108.8	2.3	3.8	3.4	3.3	2.4	2.7	0.2	0.2
BP45	33.5	4.2	1.3	0.7	2.2	1.7	0.4	0.3	0.1
DP15	53.2	3.2	2.9	2.5	1.5	3.5	1.0	0.4	0.1
DP30	24.7	4.2	2.1	1.3	0.8	3.4	2.2	0.3	0.3
DP45	7.5	4.7	0.3	2.6	2.7	0.8	2.2	0.2	0.2
PS	46.1	2.0	3.6	3.5	1.1	4.6	0.6	0.2	0.1
PP	59.3	3.7	4.1	4.3	3.4	0.7	0.8	0.2	0.2
CR	51.9	2.1	2.2	2.7	0.3	1.2	3.4	0.5	0.3
PC	21.3	3.1	2.8	3.8	1.0	2.0	2.5	0.3	0.1

### Workability

**Fig. 5:**
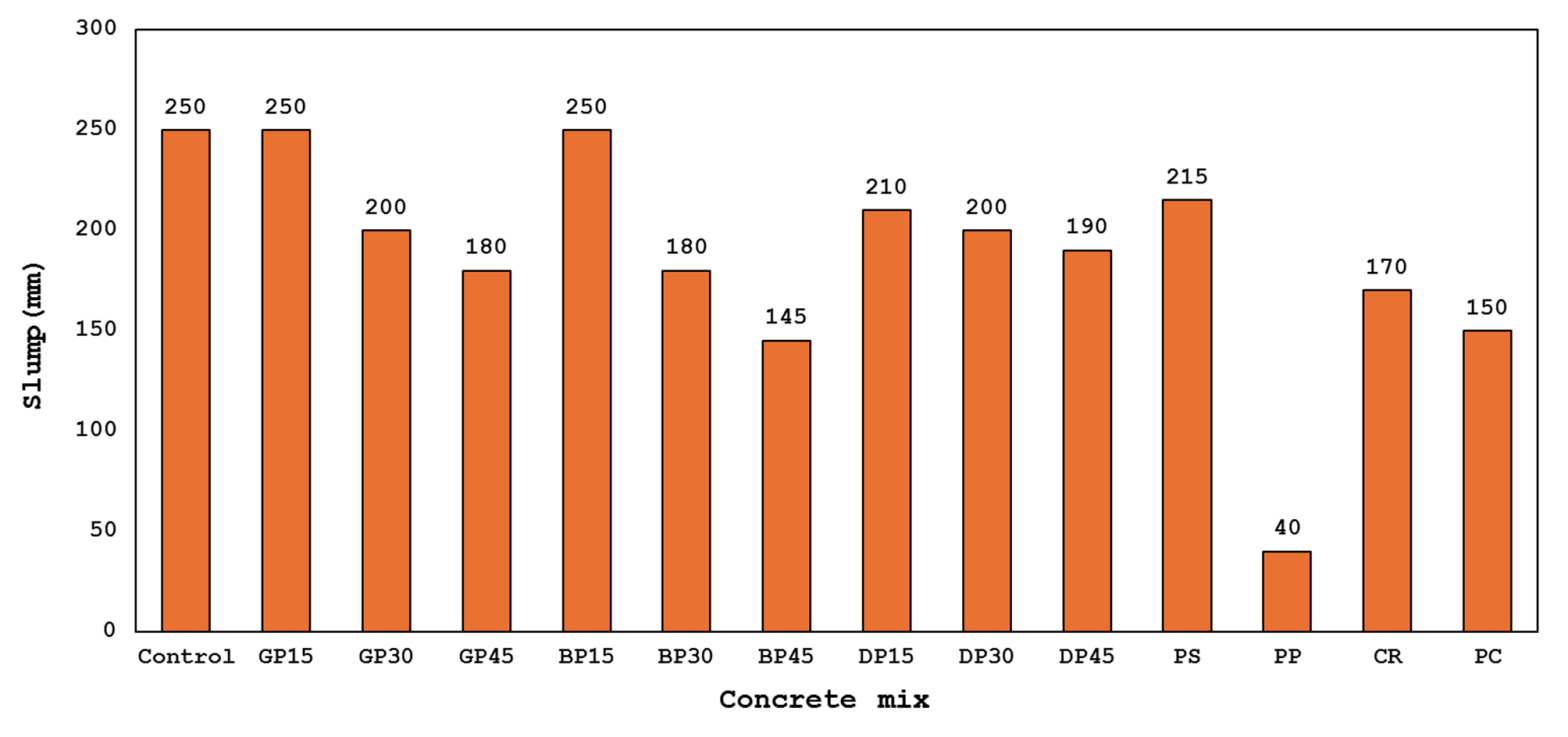
Slump value of all concrete mixes.

According to the test results shown in Fig. 5, the control mix exhibited a slump value of 250 mm, whereas the slump values of the other mixtures varied according to the waste material utilized. The slump values decreased by 20% and 28% when replacing FA with GP at 30% and 45%, respectively, in comparison to the control mixture. When BP replaced 30% and 45% of FA, the workability decreased by 28% and 42%, respectively, in comparison to the control mixture, while the slump value in HGC containing GP and BP remained constant at a replacement rate of 15% of FA. Workability decreased by 16%, 20%, and 24%, respectively, when DP replaced 15%, 30%, and 45% of the FA. The reason for the decrease in workability is due to the increased viscosity or flow resistance of the mixture, which led to a reduction in slump value. Additionally, the waste particles used (GP, BP, and DP) have a rougher and more angular shape, meaning they are irregular compared to the spherical shape of FA particles. This increases friction within the matrix, and consequently, the increased content of these waste particles results in a proportional reduction in workability.

When CR was used to replace 25% of the fine aggregate, the workability decreased by 32% in comparison to the control. This reduction in workability is primarily due to the increased irregular shape with slightly jagged edges, water absorption, and rough surface of CR in comparison to natural sand particles. Replacing 25% of the sand with PS led to a 14% decrease in workability compared to the control mix. This can be ascribed to increased irregular shape with slightly jagged edges, water absorption, and rough surface of PS in comparison to natural sand particles. PP was used to replace 25% of the sand and showed 84% less workable mix than that of the control mix. This decrease is exacerbated by PP, a polymer composed of polythene, which absorbs mixing water and induces agglomeration of the substance.

The traditional PC mix showed a workability that was 40% lower than that of the corresponding control mix. This drop was likely attributed to reduced water demand in the geopolymer matrix, resulting in decreased ettringite formation, along with the lubricating effect of sodium silicate that enhances the flowability of geopolymer concrete.

### Unit weight

**Fig. 6:**
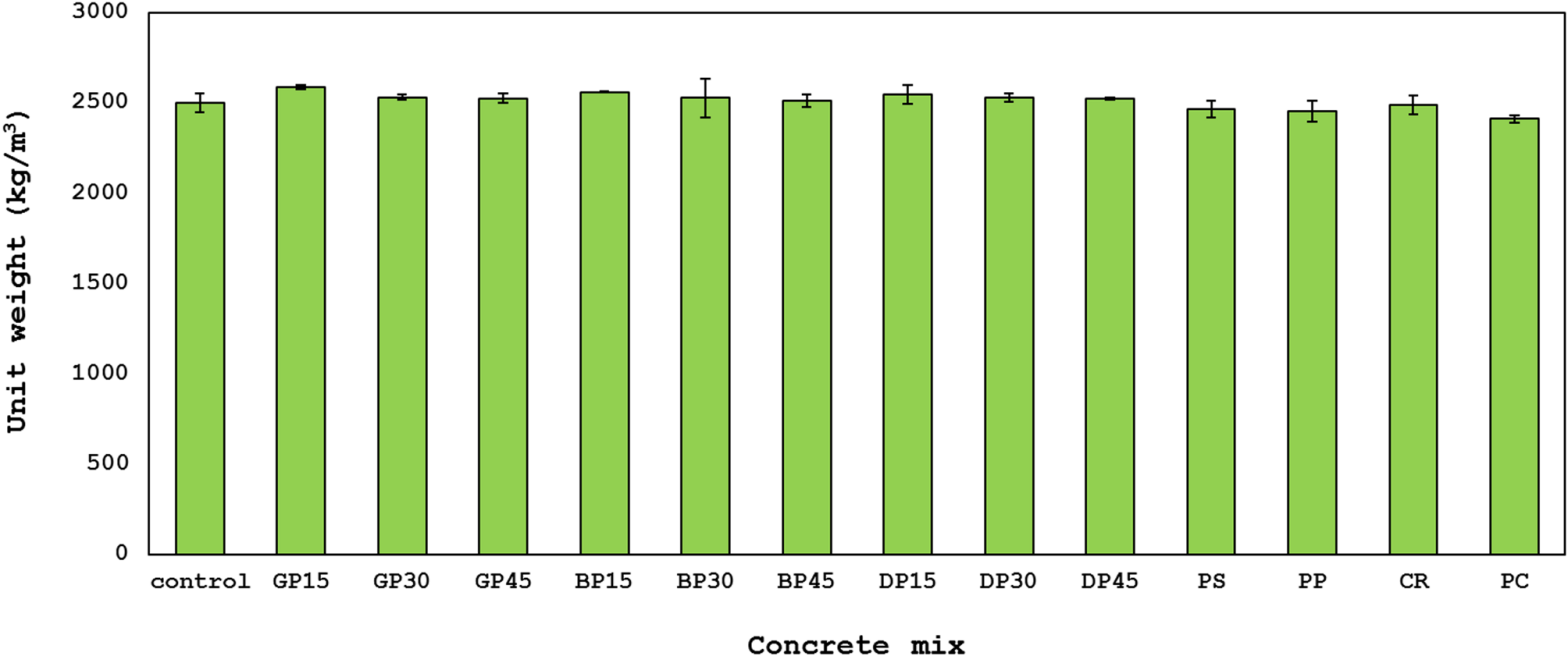
Unit weight of all concrete mixes.

The unit weight of the control mixture was 2500 kg/m³, as shown in Fig. 6. The partial replacement of FA with different waste materials, specifically GP, BP, and DP, at levels of 15%, 30%, and 45%, showed unit weights ranging from 2588 kg/m³ to 2512 kg/m³, showing a 1.54% increase for all mixes over the control mix. The primary cause of this increase is the higher specific gravity of the materials utilized in comparison to FA. In addition to exhibiting pozzolanic qualities by combining with calcium hydroxide to produce more cement-like compounds, these waste materials also react more efficiently than FA, which causes a modest increase in density.

Replacement of sand with various waste materials, namely PS, PP, and CR, at a rate of 25% results in unit weights between 2491 kg/m³ and 2452 kg/m³, indicating a reduction of 1.23% for all mixtures in comparison to the control mix. The principal reason for these decreases is the lower specific gravity of the materials utilized relative to sand.

### Compressive strength

**Fig. 7:**
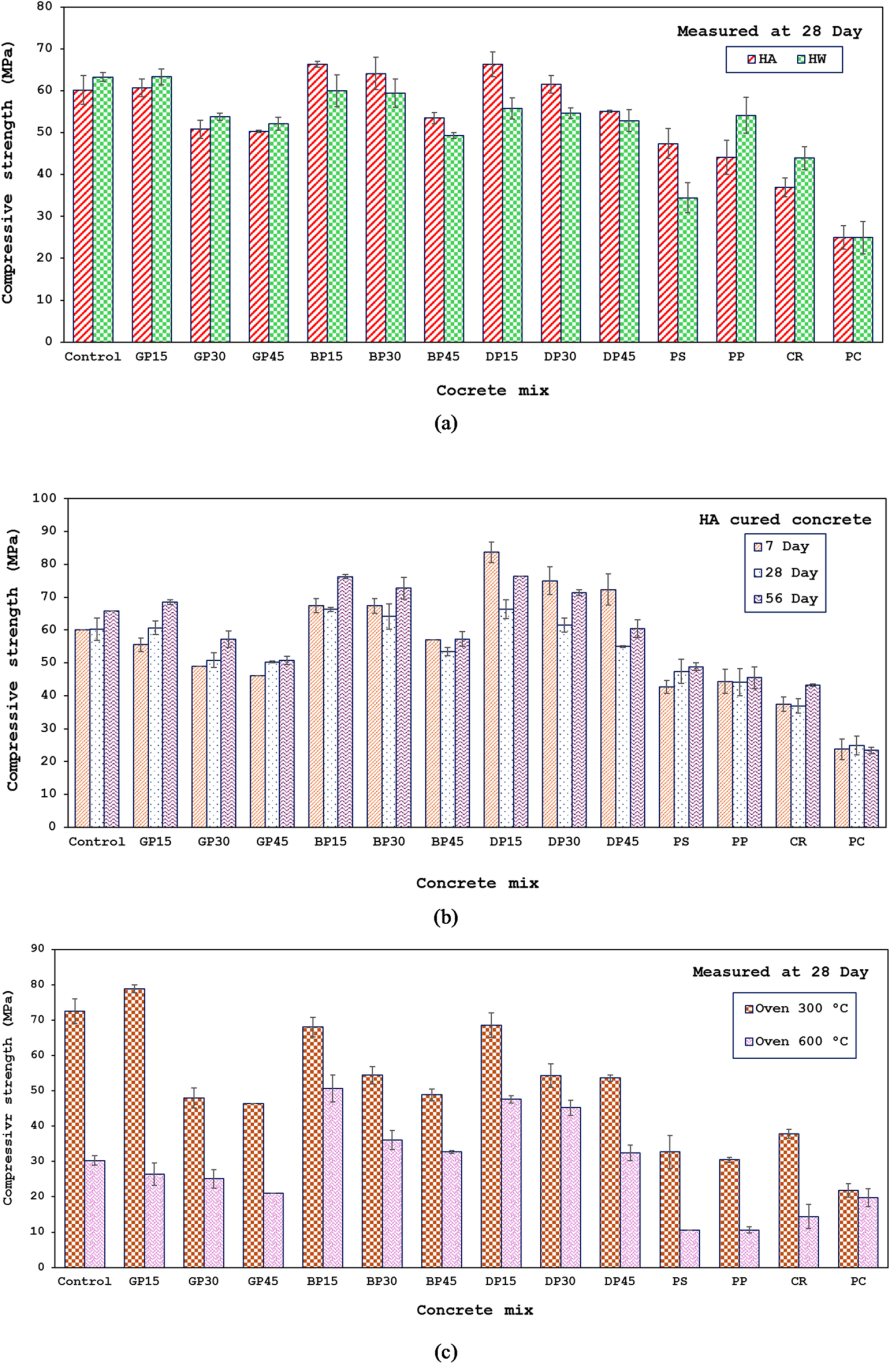
Compressive strength at: (**a**) Different curing conditions, (**b**) different concrete ages, and (**c**) different elevated temperatures.

The 28-day compressive strength under various curing conditions (HA or HW) was plotted and measured. in Fig. 7. Under HA curing conditions, no notable alteration in compressive strength was seen when 15% of the FA was substituted with GP; however, compressive strength decreased by 15.6% and 16.5% when the replacement levels increased to 30% and 45%, respectively. The reason for this is that GP has less reactive and inert characteristics. It is a filler material that effectively fills many gaps to create a dense, compact matrix. As a result, it improves compressive strength while using little replacement, however, the strength of the mortar and concrete was negatively affected if the replacement ratios increased as noticed in the 30% and 45% ratios. This is because the addition of GP in large quantities may have increased the aggregate mix’s specific surface area, requiring an excess of binding material. When the amount of GP increases and the amount of binder material decreases, more solution is needed to increase workability. This means that when there is less binder material compared to GP, more solution is required to make the mix workable, which leads to a higher binder ratio that can weaken the concrete and result in a poorly compacted mix. Similarly, the compressive strength increases by 10% when 15% of the FA is substituted with BP and DP, representing the maximum reported strength values. Additionally, the compressive strength increased by 6.6% when BP was substituted for 30% of the FA. Furthermore, replacing 30% of the FA with DP results in a 2.2% increase in compressive strength. While the replacement increased, the compressive strength decreased. When FA was replaced by 45% with DP and BP, the compressive strength decreased by 8.6% and 11%, respectively. This is consistent with findings reported by previous research^64,69–73^.

Replacing 25% of the sand volume with CR reduced the 28-day concrete strength by 38.7%. Because CR has a poor hydrophilicity, it hinders the ability of binder paste to permeate the rubber surface. This results in low adhesion at the binder/rubber interface, which increases the weak zones within the concrete matrix and lowers compressive strength. It is noteworthy that the concrete’s compressive strength may benefit from the fineness modulus of the CR employed, as it was greater than that of the sand that was substituted. Youssf et al.^74^ investigated how the CR fineness modulus affected the compressive strength of concrete and found that the higher the total fine aggregate’s fineness modulus, the higher the realized compressive strength—or, to put it another way, the lower the compressive strength losses. Substituting 25% of the sand volume with PS and PP decreased the 28-day concrete strength by 21% and 27%, respectively. The decrease in compressive strength is due to the water-repelling nature and smooth surface of plastic aggregates, which results in weak bonding between the plastics and the geopolymer matrix. This behavior was also observed in previous studies utilized plastic aggregates in cement-based composites^47–76^. Under the same conditions, concrete containing industrial waste has more than double the strength of conventional cement concrete (PC mix), highlighting the importance of using such concrete in building construction because it reduces carbon emissions, preserves the environment, and saves energy.

Figure 7(a) shows the measured 28-day compressive strength under various curing conditions (HA or HW). Comparing the HA curing method, all mixes developed less strength under the HW curing condition, except Control, GP15, GP30, GP45, PP, and CR mixes (5%, 4.4%, 6%, 3.6%, 23%, and 19% higher strength, respectively). The decrease in strength can be attributed to the higher percentage of calcium dioxide, which may have resulted in shrinkage cracks due to moisture loss at comparatively high temperatures.

Figure 7(b) shows the evolution of compressive strength for all mixes up to 56 days measured under HA curing conditions. After 7 days, the compressive strength of the mixes containing GP with 15%, 30%, and 45% replacement rates of FA decreased by 7.5%, 18%, and 23%, respectively, in comparison to the control mix. The compressive strength increased by 12% when replacing 15% and 30% of FA with BP, while the replacement ratio increased to 45%; however, the strength reduced by 5%. Replacing 15%, 30%, and 45% of FA with DP led to an increase in compressive strength by 39.5%, 25%, and 21%, respectively. According to these findings, HGC mixes can be used in construction applications since they can either attain up to 85% of their maximum strength or provide higher strength at earlier ages.

The compressive strength of mixes containing PS, PP, and CR materials declined over a period of 7 to 56 days, according to the findings of the measurements. In comparison with the control mixture, the decreases in 7 days were 29%, 26%, and 38%, respectively, and at 56 days, they were 26%, 31%, and 34%, respectively. The cause of the strength reduction is attributed to the weak adhesion between the components and the geopolymer matrix. The compressive strength of mixes containing PS, PP, and CR materials declined over a period of 7 to 56 days, according to the findings of the measurements. In particular, compared to the control mix, the decreases at 7 days were 29%, 26%, and 38%, respectively, and at 56 days, they were 26%, 31%, and 34%, respectively. The cause of the reduction is the weak adhesion between the components and the geopolymer matrix.

Compressive strength measurements for all combinations over a 28–56-day period revealed that most mixtures either increased or remained constant in strength. Specifically, the increases were 9%, 13%, 12.5%, 15%, 13.4%, 7%, 15.2%, 16%, 10%, 3%, 3.1%, and 17% for the mixes control, GP15, GP30, BP15, BP30, BP45, DP15, DP30, DP45, PS, PP, and CR, respectively, compared to their compressive strength at 28 days, while the compressive strength remained unchanged in the GP45 mix.

**Fig. 8:**
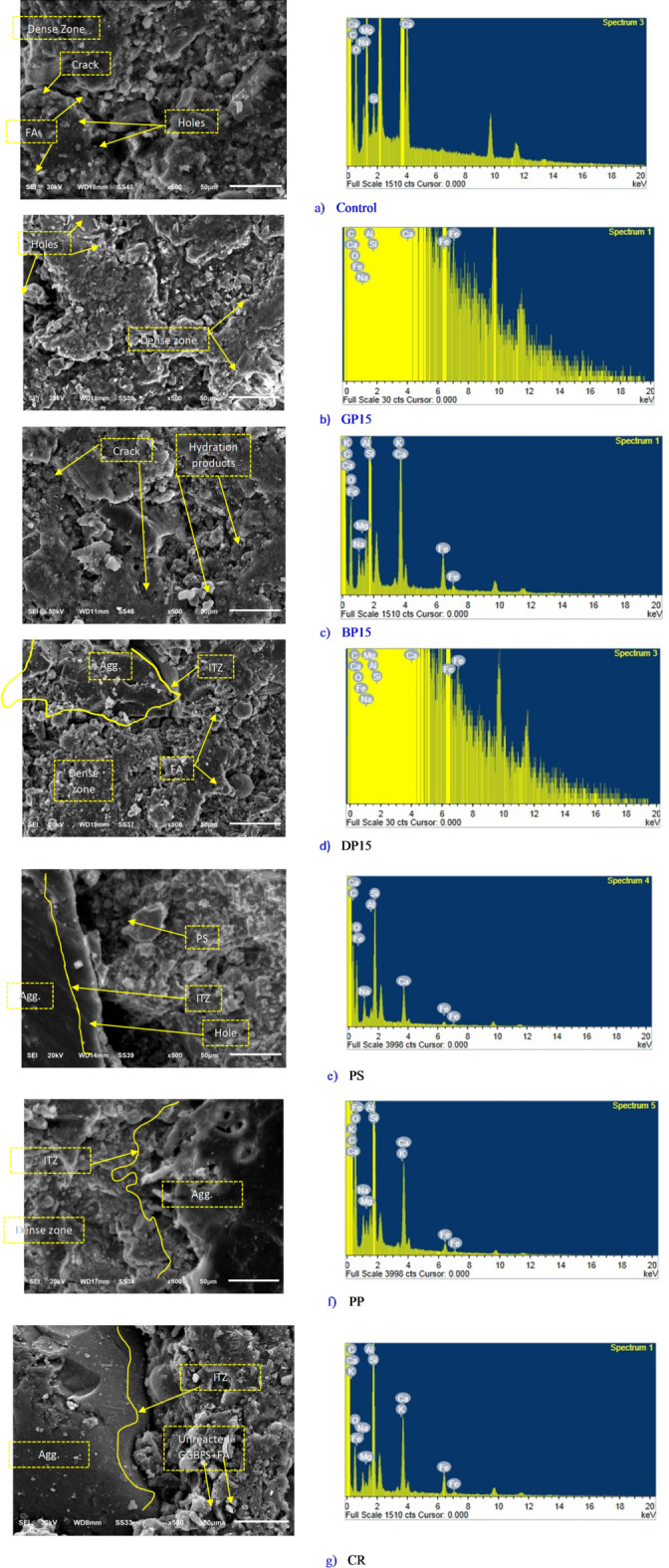
SEM (left) and EDX (right) analyses for selected HGC mixes.

The compressive strength of the HGC specimens exposed to increased high temperatures is shown in Fig. 7(C). At a temperature of 300 °C, the compressive strength of the control mix and the GP15, BP15, DP15, CR mixes increased by 20.5%, 30%, 2.6%, 3.4%, and 2.4%, respectively, compared to their strength at 28 days with the HA treatment. Exposure to moderate heat (300 °C) may facilitate additional geopolymerization or the removal of residual moisture, which leads to pore closure and increased matrix cohesion, as supported by previous studies^13,77^. However, the compressive strength of the GP30, GP45, BP30, BP45, DP30, and DP45 mixes went down. This study shows that adding more GP, BP, or DP weakens the strength because these materials are mainly used to fill small gaps and make the mixture denser. Additionally, they increase porosity, which reduces workability. Compressive strength also decreased in the PS and PP mixtures, indicating that the plastic used is incapable of withstanding fire temperatures, leading to ignition and reduced compressive strength. By comparing the HGC mixtures with the conventional PC mixture, the PC mixture does not withstand elevated temperatures like the HGC mixtures. This indicates the efficiency of HGC in withstanding high temperatures. Exposure of the specimens an elevated temperature of 600 °C resulted in a reduction of compressive strength across all mixes, illustrating that higher temperatures than 300 °C compromise compressive strength of HGC. At 600 °C, the compressive strength of all mixes decreased significantly. This decline is likely due to the dehydration of gel phases, such as N-A-S-H and C-A-S-H gels, and the breakdown of the geopolymer network. High temperatures cause the chemically bound water within these gels to be released, resulting in microstructural weakening and the development of microcracks, as suggested by both SEM imagery and EDX results (see Fig. 8; Table 6). These observations are consistent with established mechanisms described in the literature^17,77^.

Results align with findings reported by Sarker et al.^77^, who noted that fly ash-based geopolymer concrete often exhibits increased strength after moder^78^ate thermal exposure due to further geopolymerization and densification, but suffers strength loss at higher temperatures due to extensive dehydration and thermal cracking. Similarly, Tayeh et al.^17^and other studies^42^ have found that moderate heat exposure can enhance strength, while exposure above 400–600 °C leads to significant deterioration in mechanical properties.

### Flexural strength

**Fig. 9:**
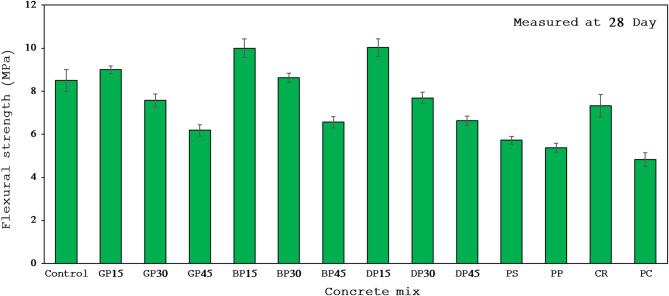
Flexural strength of all concrete mixes.

One of the key mechanical characteristics of concrete is its flexural strength, which indicates how well concrete resists bending loads, like those encountered by beams and slabs. Compressive strength and flexural strength are usually closely correlated, the higher the compressive strength the higher the corresponding flexural strength. The average flexural strengths of all mixes, which range from 4.83 to 10 MPa, are shown in Fig. 9 following 28 days of HA curing. These findings are consistent with earlier research showing that geopolymer concrete can provide higher flexural strength than cement-based concrete^77^.

All mixtures treated in HA had lower flexural strength than the control mix, except for mixes GP15, BP15, BP30, and DP15, which had flexural strength increases of 6%, 18%, 2%, and 18%, respectively. The reason for this decline is its low pozzolanic activity, increased porosity, reduced microstructural degradation, and delayed strength development. HGC exhibits superior bending resistance compared to cement-based concrete, indicating the importance of utilizing HGC in structural elements subjected to bending, such as beams and slabs, as it can endure greater bending forces, thereby enhance the longevity of the structural element and protect the reinforcement.

### Splitting tensile strength

**Fig. 10:**
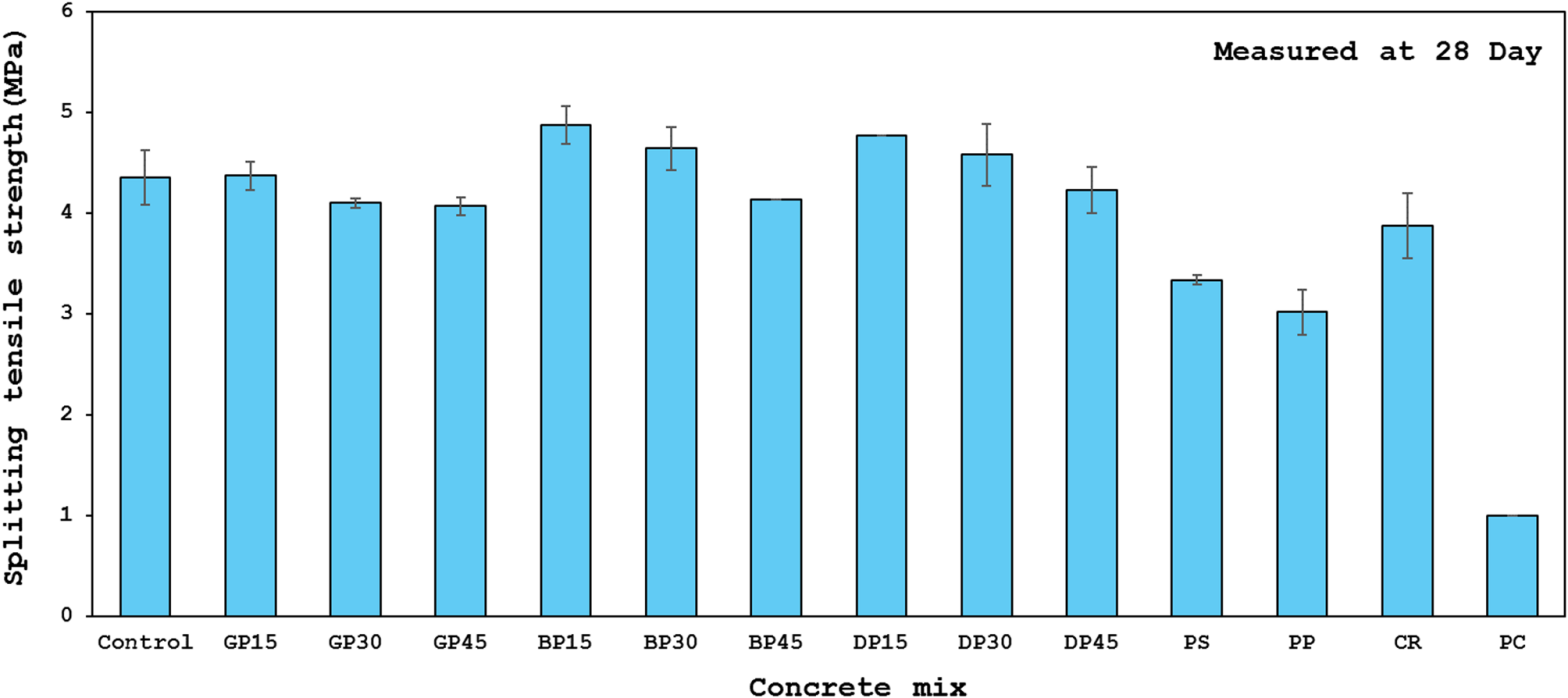
Splitting tensile strength of all concrete mixes.

One important characteristic that affects concrete behavior is its splitting tensile strength, specifically about shear resistance, crack initiation, and propagation. Calculating splitting tensile strength is crucial for the integrity of the structure since concrete is brittle and weak under tension. Table 4; Fig. 10 show the splitting tensile strength results for all mixes at 28 days under HA treatment settings. The values range from 4.88 to 1.05 MPa. About 7% of the compressive strength is the average splitting tensile strength. At 28 days, the control mix’s average splitting tensile strength was 4.36 MPa. Compared to the control mixture, there are increases in splitting tensile strength in the GP15, BP15, BP30, DP15, and DP30 mixtures. Improvements in compressive strength and splitting tensile strength are consistent, see Fig. 7(a). This is explained by the alkali-pozzolanic reaction’s higher efficiency, the components’ better internal distribution, and the molecules’ stronger bonds. Other mixtures illustrated reductions in splitting tensile strength, showing that higher replacement ratios of different waste materials adversely impact splitting tensile strength.

From the splitting tensile strength results, it can be concluded that HGC is stronger in tension compared to conventional cement-based concrete, showing the importance of using eco-friendly concrete in parts of buildings that are likely to crack, like beams and slabs, because it can handle more tension, which helps make the structure last longer and protects the reinforcement.

### Microstructure analyses

Figure 8 presents the morphology surface of selected mixes namely, Control, GP15, BP15, DP15, PS, PP, and CR. It is evident from Fig. 8 (a) that the surface contains numerous pores and cracks, with only a few dense zones. This could be attributed to the high content of FA in this mix, which was cured in HA conditions. Consequently, it may have been more susceptible to cracking. Moreover, Figs. 8 (b to d), reveal comparatively denser and more cohesive zones than the control mix. This is because of the existence of GP, BP, and DP, which act as filler materials. These fillers reduce the formation of pores seen in the control mix, thereby enhancing the overall mechanical properties of these mixes. This confirms the correlation between the morphological surface of the fractured specimens and their mechanical properties. Additionally, in these three mixes (containing GP, BP, and DP), the interfacial transition zone (ITZ) appears to form a relatively strong region. This is due to the presence of effective hydration products near this zone, resulting from two main factors: the strong interaction between the activator and the geopolymeric materials, and the positive effect of these fillers, which enhance the bond between the aggregate and the geopolymer paste. As for images in Figs. 8 (e to g), the ITZ is clearly more pronounced, forming a sharp boundary between the geopolymer paste and the aggregates. This can be attributed to the high absorptivity of PS and PP materials, as indicated by the slump test, which causes uneven coverage of the aggregates by the geopolymer paste. As a result, hydration products are not effectively formed in this region, which explains the reduction in compressive strength.

Figure 8 shows the distribution of chemical elements detected by the EDX analysis. Table 6 clearly demonstrates that as the Ca/Si ratio increases, the compressive strength decreases, and vice versa. This indicates a general correlation between microstructural characteristics and mechanical properties, validating the significance of these tests and their interrelation. Additionally, the elements silica (Si) and alumina (Al) play a crucial role in forming dense regions and reducing the presence of pores and cracks, as shown in the EDX analysis results.

**Table 6:** EDX elements for the selected mixes.

Element		C	O	Na	Mg	Al	Si	K	Ca	Fe	Ti	Ca/Si
Control	Weight%	57.82	32.22	1.89	0.54	0.93	2.80	0.14	2.62	0.97	0.08	0.94
	Atomic%	67.29	28.15	1.15	0.31	0.48	1.40	0.05	0.91	0.24	0.02	
GP15	Weight%	38.25	41.31	3.69	-	0.65	11.04	-	4.37	0.70	-	0.40
	Atomic%	49.25	39.94	2.48	-	0.37	6.08	-	1.69	0.19	-	
BP15	Weight%	62.07	25.45	1.59	0.72	0.97	4.78	0.14	3.04	1.24	-	0.64
	Atomic%	72.13	22.20	0.97	0.41	0.50	2.38	0.05	1.06	0.31	-	
DP15	Weight%	29.37	44.78	3.47	3.33	1.47	6.40	-	9.94	1.24	-	1.55
	Atomic%	40.18	46.00	2.48	2.25	0.90	3.75	-	4.07	0.36	-	
PS	Weight%	51.80	33.36	2.25	-	0.66	7.72	-	3.38	0.83	-	0.44
	Atomic%	52.56	30.24	1.42	-	0.35	3.99	-	1.22	0.22	-	
PP	Weight%	75.87	17.53	0.58	0.30	0.37	3.12	0.09	1.70	0.43	-	0.54
	Atomic%	82.81	14.36	0.33	0.16	0.18	1.46	0.03	0.55	0.10	-	
CR	Weight%	62.15	25.27	1.53	0.41	0.92	5.83	0.15	2.48	1.26	-	0.43
	Atomic%	72.19	22.04	0.93	0.23	0.47	2.89	0.05	0.86	0.32	-	

## Conclusions

This research focused on assessing the combined effect of GP waste, BP waste, DP waste, CR, and PW in the production of HGC. GP, BP, and DP were used as partial replacements for geopolymer binder (FA) at levels of 15%, 30%, and 45%, Additionally, at a level of 25%, CR and PW were utilized as partial substitutes for sand. Two different forms of PW were used: plastic shales (PS) and plastic pellets (PP). The performance of the introduced HGC was assessed through several measurements including compressive strength, splitting tensile strength, and flexural strength. The compressive strength was measured under various curing conditions such as heat followed by air (HA) and heat followed by water (HW), as well as under high elevated temperatures (room temperature, 300 ᵒC, and 600 ᵒC). SEM and EDX spectroscopy were used for analyzing the microstructure of a few chosen HGC mixtures. This study’s primary findings are summed up as follows:

1. HGC with a binder content of 521 kg/m³, an AA/Binder ratio of 0.40, an SS/SH ratio of 1.70, and a 12 M molarity achieved the highest compressive strength of 76.4 MPa under HA curing conditions after 56 days.
2. Workability decreased by 27% when FA was replaced with waste materials and 32%, 14%, and 84% when 25% of geopolymer concrete sand volume was replaced with CR, PS, and PP.
3. The GP15, BP15, and DP15 mixes showed improved performance after 28 days of curing in HA conditions, with high compressive, tensile, and flexural strengths and denser, more uniform microstructures, suggesting potential use in sustainable building.
4. The BP content, up to 30%, achieved a high compressive strength of 64.13 MPa, flexural strength of 8.63 MPa, and tensile strength of 4.65 MPa after 28 days under HA curing conditions.
5. After 28 days of HA curing, replacing 30% and 45% of FA with GP reduced compressive strength by 15.6% and 16.5%, splitting tensile strength by 6% and 7%, and flexural strength by 11% and 27%, compared to the control mix.
6. Replacing 45% FA volume with BP and DP reduced compressive strength, flexural strength, and tensile strength by 11%, 8.6%, 23%, and 22%, 5%, and 3%, respectively, under HA curing conditions.
7. The study found that replacing 25% of sand volume with CR, PS, and PP reduced compressive strength, flexural strength, and splitting tensile strength after 28 days of HA curing. These compositions produced less dense and more irregular microstructures with more voids, as confirmed by SEM and EDX analyses.
8. At 300 °C, the Control, GP15, BP15, DP15, and CR mixes all showed higher compressive strength than under normal conditions, with values of 72.5 MPa (Control), 78.8 MPa (GP15), 68 MPa (BP15), 68.53 MPa (DP15), and 37.8 MPa (CR). However, compressive strength for all mixes decreased when exposed to 600 °C.
9. In comparison with HA curing, all the mixtures exhibited reduced strength under the HW curing, except for the control mix, GP mixes, PP, and CR.

Overall, the comparison with the traditional concrete mix made from Portland cement showed that all hybrid geopolymer concrete mixes exhibited better mechanical properties. According to the result, hybrid geopolymer concrete can be used as an alternative for traditional concrete in structural applications.

## Recommendations for future research

This study suggests several future research directions, based on its encouraging results. This includes durability measurements such as permeability, sulfate or chloride resistance, freeze-thaw stability, and shrinkage. In addition, life cycle analysis and carbon dioxide emissions analysis are recommended for future studies.

## Data Availability

All data generated or analyzed in this study are included in this manuscript.
